# Synergic Benefits of Air Pollutant Reduction, CO_2_ Emission Abatement, and Water Saving under the Goal of Achieving Carbon Emission Peak: The Case of Tangshan City, China

**DOI:** 10.3390/ijerph19127145

**Published:** 2022-06-10

**Authors:** Rupu Yang, Min Wang, Mengxue Zhao, Xiangzhao Feng

**Affiliations:** Policy Research Center for Environment and Economy, Ministry of Ecology and Environment of the People’s Republic of China, Beijing 100029, China; yang.rupu@prcee.org (R.Y.); wang.min@prcee.org (M.W.); zhao.mengxue@prcee.org (M.Z.)

**Keywords:** synergic benefits, LEAP model, CO_2_ emission, air pollutants, water consumption, carbon emission peak

## Abstract

The study aims to explore the synergic benefits of reducing air pollutants and CO_2_ and water consumption under the carbon emission peak (CEP) policies at a city level. Air pollutants and CO_2_ emissions are predicted by the Low Emissions Analysis Platform (LEAP) model, and the water consumption is forecast by the quota method. Two scenarios are constructed with the same policies, but to different degrees: the reference scenario achieves CEP in 2030, and the green and low carbon scenario achieves CEP in 2025. The prediction results show that air pollutant emissions, CO_2_ emissions, and water consumption can be obviously decreased by intensifying the CEP policies. The synergic abatement effect was illustrated by the synergic reduction curve. Accelerating the adjustment of economic structure saves the most water, reduces the greatest amount of CO_2_ emission, and also obtains the best synergic reduction capability between water consumption and CO_2_ emission. Transforming the traditionally long process of steelmaking toward a short electric process reduces the majority of PM_2.5_, SO_2_, and VOC emissions, while consuming more water. The study provides a new viewpoint to assess and optimize the CEP action plan at city levels.

## 1. Introduction

Anthropogenic activities have led to severe worldwide environmental issues, and the global warming caused by increasing greenhouse gas emissions is regarded as an urgent challenge for human sustainable development. To contribute to building a community with a shared future for mankind, China has pledged to achieve carbon emissions peak (CEP) before 2030 and carbon neutrality by 2060 [1], and a so-called “1+N” policy framework at national level was launched to guarantee the targets in 2021. CEP has become the priority task for governments at all levels.

China has devoted enormous efforts to control carbon emissions, such as the particularly rigorous “double control” in energy consumption since the 12th Five-Year Plan. It achieved remarkable results with the carbon emission intensity decreasing 48.4% in 2020 compared with the levels of 2005 [2]. Air pollutants were reduced synergistically. During the 13th Five-Year Plan period, 87% of the days in cities at the prefecture level and above had good or excellent air quality. The average concentration of fine particulate matter (PM_2.5_) in cities at the prefecture level and above that did not meet the standard fell 28.8 percent from 2015 [3]. However, numerous cities still face the problem of air pollution, especially the “2 + 26” cities in the Jing-Jin-Ji region, and the marginal benefits of continuing the end-of-pipe treatment are decreasing rapidly. The CEP target provides a great opportunity to improve the ecological environment through cracking sources and structural problems.

Studies regarding the synergic reduction effects between air pollutants and greenhouse gas (GHG) started in the late 1990s, and co-control measures and the corresponding synergic benefits have been widely discussed since then [4,5]. In 2004, the common sources of air pollutants and carbon emission were identified [6]. This research ranges from different countries to regions and various industry sectors. The co-benefits of reducing CO_2_ and SO_2_ emissions in Thailand were examined over a variety of policies in different industry sectors [7]. A series of policy options were recognized to achieve the co-benefits of air quality improvement and GHG mitigation in the Seoul metropolitan area via a cost-effectiveness analysis [8]. It was found that the synergic reduction in hydrocarbon, NOx, PM, etc. with CO_2_ has a considerable potential by implementing this energy-saving policy in the Chinese transportation sector [9]. To compare the synergic effects of various policies, Mao et al. [10] proposed the co-control effects coordinate system by setting the reduction effect of air pollutants as X-ray and the reduction effect of CO_2_ as Y-ray. Those points located in the first quadrant suggest corresponding policies that have positive synergic reduction effects, and the larger the angle, the better the synergic reduction ability. There are several explorations of co-benefits in the transportation sector in different cities in China. Adjusting transport modes and increasing the application of electricity are the two most effective measures in Guangzhou [11]. Additionally, shifting transportation modes are the most promising policies in the short term and electrification takes a more important role in the long term in Chongqing to reduce air pollutants and CO_2_ synergistically [12].

Synergistically reducing CO_2_ and air pollutant emissions has already been recognized as one of the key aspects to implementing the CEP plan, but water saving issues have rarely been investigated in the context of the CEP. Water consumption is among the priority concerns in high-quality development in China, and a series of top-level regulations have addressed the importance of saving water toward the target of CEP and carbon neutrality [13,14,15]. Especially in the Yellow River basin, water resources are relatively scarce compared with the south-eastern regions.

Water consumption has a close relationship with fossil fuel consumption since it usually acts as the energy carrier to transform chemical/thermal energy into mechanical energy or to be a heat transfer medium. The synergic effects of water-saving potential and CO_2_ emission reduction are usually accompanied by the keywords of water–energy nexus [16,17] or water–energy–carbon nexus [18,19] as in the literature. The water–carbon nexus system in the Jing-Jin-Ji region has been studied through a multivariate statistical approach, and various industrial technology upgrade policies were simulated [20]. It was found that heavy industry in Hebei Province and electricity generation and heating industry in Beijing are the two most critical sectors in water–carbon management (i.e., water consumption intensity and CO_2_ emission intensity would decrease by 3.3% and 15.3%, respectively). The energy–water–carbon nexus in the Yangtze River Delta has also been explored, and there is a significant potential for water saving and carbon reductions as well as system efficiency improvement [21]. The water–energy–carbon nexus was investigated in a power plant, in which considerable synergic benefits can be achieved through energy-saving techniques [22]. The application in combined heat and power systems revealed that water-saving potential and CO_2_ emission reductions can be up to 15.5% and 67.5%, respectively, by optimizing thermal storage [23]. The water–energy–food nexus is another important topic in the discussion of the synergic benefits of water saving and carbon reductions for croplands that attracts much research attention [24,25]. There is also research dedicated to specific industries; a case study from a plant level of China’s iron and steel industry found that reductions of 17.4% of fresh water and 37.4% of CO_2_ emissions can be achieved by implementing 31 energy-saving technologies [26].

Reviewing the literature, the synergic reduction benefits between CO_2_ and air pollutants have been widely discussed; the CO_2_–water saving relationship has also been studied at various levels. However, air pollutant reductions, CO_2_ abatement, and water saving have rarely been discussed by the state-of-the-art literature, and is of great significance under the carbon emission control background, since it will contribute considerably to those regions with an urgency to optimize the CEP plan and also the carbon neutrality strategy.

This work aims to explore the multi-benefits of reducing air pollutants, CO_2_ emissions, and water consumption when implementing the CEP plan through a bottom-up method by taking Tangshan City (the Chinese city famous for its iron and steel industry) as an example. Additionally, the Synergic Reduction Curve (SRC) is proposed to illustrate the priority of various policies in terms of the synergic reduction capability, which can help policymakers to understand the potential contribution of individual measures more directly.

## 2. Materials and Methods

### 2.1. LEAP Model

LEAP was developed by the Stockholm Environment Institute to act as an efficient tool to carry out energy policy analysis, environment performance simulation and climate change mitigation assessment. It is a bottom-up, integrated, and scenario-based model that can predict energy consumption of end-use sectors and the corresponding airborne emissions, and it suits different scales ranging from cities and states to national, regional, and global aspects. At least 32 countries used LEAP to create energy and emission scenarios that were the basis for their Intended Nationally Determined Contributions on Climate Change. It has also been adopted in various cities in China [27,28]. Nevertheless, LEAP is seldom applied in the study of air pollutant emission since the accounting system lacks an emission factor. In the present work, the customized LEAP-Tangshan (LEAP-TS) model is developed through the sectoral activity and emission data compilation by taking the Technology and Environmental Database (TED) as an emission factor reference [12,29].

The LEAP-TS model was built from field research, and it contains three modules: Key Assumptions, Demand, and Transformation. Population, urbanization ratio, gross domestic product (GDP) growth, and economic structure are part of the Key Assumptions. Demand refers to energy end-use sectors, including agriculture, secondary industries, construction, transportation, services, and households. Transformation corresponds to the electricity and heat supply in Tangshan, which is mainly combined with coal and heat power plants. To decarbonize the energy supply, the local government decided to accelerate the development of solar and wind energy. The activity and energy structure of the model is provided in Table 1.

The LEAP-TS predicts the CO_2_ and air pollutant emissions based on the sector specific activity data under various source-governance policies, as provided by Equation (1).

(1) $$E_{CO_{2}}=\sum_{i}\sum_{j}A_{i,j}\times \alpha_{i,j}^{CO_{2}}$$

where $A_{i,j}$ represents the activity level of the i sector on j fuel consumption (9 kinds of fossil fuels according to the investigation) and $\alpha_{i,j}^{CO_{2}}$ indicates the CO_2_ emission factor of *j* fuel consumption in the *i* sector.

The primary emissions of PM_2.5_, NOx, SO_2_ and VOCs are obtained by Equations (2)–(5), respectively. Taking PM_2.5_ as an example, $TED_{i,j}^{PM_{2.5}}$ depicts the emission coefficient of PM_2.5_ per unit of *j* energy consumption in the *i* sector with the information of default technology and environment database in the LEAP, and $\beta_{i,j}^{PM_{2.5}}$ is the correction coefficient of PM_2.5_ emissions due to the localized condition such as various end-of-pipe removal efficiencies.

(2) $$E_{PM_{2.5}}=\sum_{i}\sum_{j}A_{i,j}\times TED_{i,j}^{PM_{2.5}}\times \beta_{i,j}^{PM_{2.5}}$$

(3) $$E_{NO_{x}}=\sum_{i}\sum_{j}A_{i,j}\times TED_{i,j}^{NO_{x}}\times \beta_{i,j}^{NO_{x}}$$

(4) $$E_{SO_{2}}=\sum_{i}\sum_{j}A_{i,j}\times TED_{i,j}^{SO_{2}}\times \beta_{i,j}^{SO_{2}}$$

(5) $$E_{VOCs}=\sum_{i}\sum_{j}A_{i,j}\times TED_{i,j}^{VOCs}\times \beta_{i,j}^{VOCs}$$

The emission reduction effect of individual policies and their combinations can be calculated under different scenarios. The CO_2_ emission reduction can be obtained by Equation (6) through the comparison with the reference scenario. The reduced air pollutants can be reached similarly, and PM_2.5_ was taken as an example in Equation (7).

(6) $$\Delta E_{CO_{2}}^{s}=\sum_{i}\sum_{j}\Delta A_{i,j}^{s}\times \alpha_{i,j}^{CO_{2}}$$

(7) $$\Delta E_{PM_{2.5}}^{s}=\sum_{i}\sum_{j}\Delta A_{i,j}^{s}\times TED_{i,j}^{PM_{2.5}}\times \beta_{i,j}^{PM_{2.5}}$$

### 2.2. The Prediction of Water Consumption and Water Saving

#### 2.2.1. The Water Consumption Prediction

**Figure 1:**
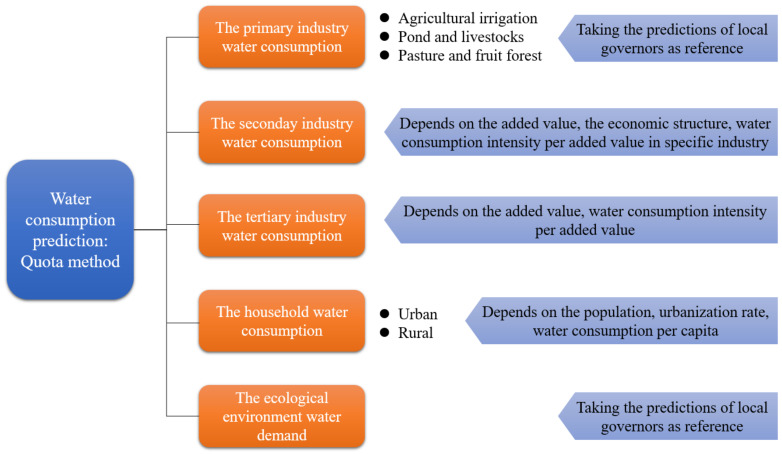
The prediction of water consumption.

The quota method was employed to estimate future water consumption as illustrated in Figure 1, which also combines the advice from the Tangshan Water Conservancy Bureau. There are mainly five parts of water consumption: the primary industry, secondary industry, tertiary industry, households, and the ecological environmental water demand. Most of water resource is consumed in the primary industry, and agricultural irrigation to be more specific. The estimation of the water consumption can either follow the bottom-up approach or by referring to the prediction of the local governors who are more familiar with the city’s condition. The latter choice was selected in this work since the emphasis was on the non-agricultural sectors. The water consumption in the secondary and tertiary industries both utilize the activity and intensity for their forecasting. The secondary industry ought to separate the high-consumption and high-emission industries from others, considering that their contribution to the economic structure varies under different development scenarios. Household water consumption is differentiated in the urban and rural parts. The water demand in the ecological environment part depends on the city construction, the ecological demand of the plant, and the river, which can also obtain solid predictions by referencing the local governors.

#### 2.2.2. The Water Saving

Reviewing the CEP plan of the state and the corresponding plan of the local government, there are four types of measures that are able to affect water consumption, as follows:

- Adjusting the economic structure to promote low-emission high-efficiency industries or products. For instance, extending the steel industry chain and developing high value-added special steel products, which can greatly reduce the water consumption per added value.
- Controlling the output of the main products of high-consumption and high-emission industries.
- Shifting toward low-emission process techniques. For example, electric furnace short steelmaking processes is much more environmentally friendly compared with the current long process technology. Additionally, the corresponding water consumption per crude steel differs considerably.
- Improving energy efficiency. The energy efficiency improvement in the secondary industry can usually lead to water savings. As an example, the cooling water demand might be reduced when more energy can be recycled.

All the above measures are part of the secondary and tertiary industries since they consumed the majority of energy and account for the major part of emissions. The water consumption variation can be calculated by Equation (8) for the first three types, via the activity changing information. Δ*P* stands for the activity variation, which can be industry added value, the quantity of products in total, and the quantity of products with specific technology. $I_{m}$ represents the water consumption intensity of the specific activity.

(8) $$\Delta Water_{1}=\sum_{m}\Delta P_{m}\times I_{m}$$

Water saving caused by energy efficiency improvements can be estimated by Equation (9). Δ*EC* depicts the saved energy consumption due to the efficiency improvement, and *η* denotes the potential water saving per tce of saved energy. To analyze the water saving potential by iterating each potential technology from a bottom-up manner might give more detailed information at the micro-level. However, it is out of the scope of this work to discuss the specific energy-saving technology, and the lumped relation of Equation (9) can also be effective from a region view.

(9) $$\Delta Water_{2}\;=\;\Delta EC\;\times \;\eta$$

A similar approach was also utilized by [30] when assessing the water-saving potential of the secondary industry. Electricity and heat are mainly supplied by the coal power plant in Tangshan, and it has a considerable potential to improve energy efficiency. The water-saving potential of the energy-saving technology in the coal power plant takes the result of [31] as a reference. The iron and steel industry are critical sectors to increase energy efficiency. There are over forty available technologies to reduce energy consumption, such as the clustering, ironmaking, steelmaking, and rolling processes. The accompanied water-saving capability takes the data of [32] as a reference. The energy efficiency improvement measures in the cement industry rely more on high-efficiency equipment, which is free of water consumption [33], and it is neglected in this work. Water consumption in the chemical industry simultaneously serves as both the ingredient and the energy carrier. Differentiating from other secondary industries, the chemical industry (especially the petrochemical industry) is shaped by hundreds of major products and process techniques. It is possible to identify the potential for energy efficiency improvement via the bottom-up method, but the involved water consumption might increase or decrease depending on the specific technology among thousands of possibilities. Considering that most of the chemical industry projects are being planned in Tangshan, water saving through energy efficiency is not be discussed in this study to avoid unpredicted bias.

### 2.3. Synergic Reduction Effect

In this study, synergic reduction intensity (*RSI*) is employed to exhibit the co-benefit of reducing specific air pollutants and CO_2_ emissions, as shown in Equation (10). The intensity indicator can reflect the reduction ability of the total amount more directly. It is consistent with the government’s total quantity control policy. *RSI* can be positive, negative, or zero. When *RSI* is negative, it indicates that the corresponding measures cannot achieve the synergic reduction, and either CO_2_ or pollutant emissions will abate while others will increase. It has to be noticed that the equation cannot be utilized when there is no variation in PM_2.5_ emissions.

(10) $$RSI_{PM_{2.5}/CO_{2}}^{s}=\frac{\Delta E_{CO_{2}}^{s}}{\Delta E_{PM_{2.5}}^{s}}$$

The reduction elasticity *ELS* is utilized to study the co-benefits of reducing multi-air pollutants and CO_2_ emissions, as shown in Equation (11), which is a common tool used in the field [11,12,34]. *ELS* is a dimensionless number, and the interpretation of the *ELS* is similar to *RSI*, but it deals with the emission reduction degree instead of the quantity. The degree comparison allows for the elimination of the absolute quantity or the reference influence and enables the analysis of the effect on various emissions from an objective point of view.

(11) $$ELS_{PM_{2.5}/CO_{2}}^{s}=\frac{\Delta E_{CO_{2}}^{s}/E_{CO_{2}}^{s}}{\Delta E_{PM_{2.5}}^{s}/E_{PM_{2.5}}^{s}}$$

The coordinate system of co-control effects proposed by [10] was widely applied in the synergic reduction in GHGs and air pollutants toward sectoral [11,34,35] and regional levels [36,37]. However, it can hardly reveal the accumulated reduction, and it might also be difficult to distinguish the contribution of various measures since they are easily gathered in a small region. In the end, it might be unable to reveal the priority of policies in terms of the synergic reduction capability. Inspired by the marginal abatement cost curve presented by Mckinsey [38], the synergic reduction curve (SRV) was proposed to help rank the synergic capability of various policies that can achieve air pollutant reductions and the abatement of CO_2_ emissions in a vivid manner.

**Figure 2:**
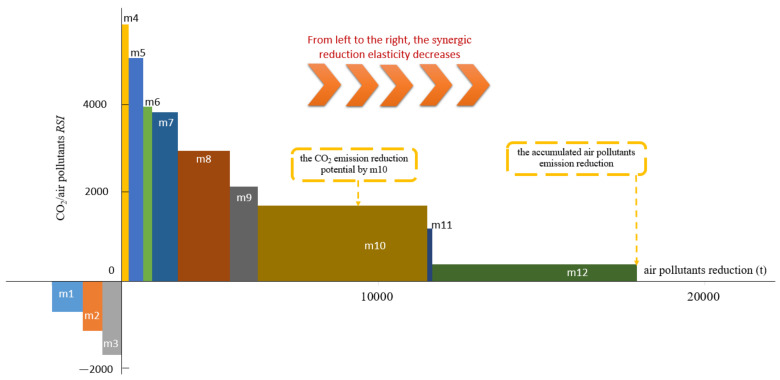
The example of the SRV.

Taking Figure 2, for instance, assume there are 12 measures to synergistically affect the air pollutants and CO_2_ emissions. The X-ray represents the air pollutants’ reduction, and the length of each block on the axis denotes the maximum air pollutant reduction capability by the corresponding measure. The Y-ray signifies the *RSI* of CO_2_; the larger the y value, the more powerful the synergic effect. For those measures located in the first quadrant (m4~m12), the *RSI* gradually decreases, and m4 exhibits the best capability of CO_2_ synergic reduction by removing air pollutants per unit. However, for measures m1~m3, they are able to reduce CO_2_ emissions, but increase the air pollutants at the same time. Similar interpretations can be applied for those measures in the second and fourth quadrants, which are not appealing to pursue the synergic effect. In other aspects, the area of each block illustrates the CO_2_ emission mitigation potential. It can be read that m12 is able to mostly reduce the air pollutant emissions, and m10 has the best ability to abate CO_2_ emissions.

### 2.4. Data Collection

Tangshan City was selected as the case study since it is under great pressure to control the CO_2_ and air pollutant emissions to achieve the CEP and air quality improvement. Tangshan is located in the eastern part of the Hebei Province and the center of Bohai Bay, located across 117°31′–119°19′ E, 38°55′–40°28′ N with an area of 1.434 million hectares. By the end of 2020, the permanent population of Tangshan reached 7.964 million, and urbanization was 64.3%. As the major industrialized city of the Jing-Jin-Ji region, Tangshan has achieved fast economic growth in recent decades. Its regional GDP reached CNY 696.4 billion in 2020 from CNY 345.3 billion in 2010 (by taking 2015 as the constant price year), which corresponds to an average annual increase of 7.3%. Tangshan is also famous for its iron and steel industries, and the secondary industry accounts for 53.2% of the regional GDP in 2020. In response to the central government’s goal of green and low carbon development, Tangshan has carried out a series of policies in recent years. For example, the “Several Provisions on the Prevention and Control of Air Pollution in Tangshan City”, “Ultra-low emission standard of air pollutants for iron and steel industry”, “Ultra-low emission standard for air pollutants in coking chemical industry” to control rigorously the air pollutant emissions. “Key points of energy-saving and coal cutting work in Tangshan City” was created for controlling CO_2_ emissions and pollutants by managing coal consumption.

Considering the data availability and integrity, the year 2018 was selected as the baseline to compile the CO_2_ emission, air pollutant emissions, and water consumption inventory. The socio-economic information and the sectoral energy consumption of various fuel types in 2018 are mainly referred to [39], as well as the energy balance table provided by the Tangshan Statistics Bureau. Gasoline and diesel consumption information came from the local Commerce Bureau. For transportation data, the freight turnover came from the statistical yearbook [39], and the number of vehicles powered by various types of fuel with different emission standards was provided by Tangshan Traffic Bureau. CO_2_ emission factors of fossil fuels involve the LHV, carbon content, and oxidation ratio. Most of them were obtained through field investigation from representative companies, such as the major coal power plants, and iron and steel, cement, chemical companies. The remainder of the reference values were taken from Guidelines for Accounting Methods and Reporting of Greenhouse Gas Emissions of Enterprises in Key Industries in the Hebei Province [40,41,42]. The major air pollutant emission factor takes the default TED data in LEAP and multiplies correction factors to be coherent with the Air Pollution Source Emission Inventory offered by Tangshan Ecological Environment Bureau.

**Figure 3:**
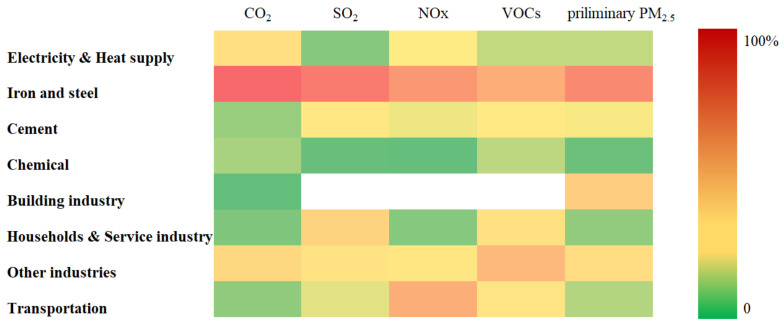
The sectoral contribution of CO_2_ and air pollutant emissions of Tangshan in 2018.

Analyzing the CO_2_ and air pollutant emissions from the sectoral aspect, iron and steel contribute the majority of CO_2_, SO_2_, NOx, VOCs, and preliminary PM_2.5_ as shown in Figure 3. In terms of CO_2_ emissions, the electricity and heat supply accounts for about 12.0%, the chemical and cement industry contributes about 2.9% and 2.3%. Except for the iron and steel industries, the household and service industries contribute the second most SO_2_ emissions, the cement and transportation industries are also critical departments to reducing the SO_2_ emissions. Transportation was responsible for 33.6% of NOx emissions, the electricity and heat supply sectors contributed 6.5% of NOx emissions. In terms of VOCs emissions, the coking industry accounts for a major part, households and service industries and transportation sectors are also important to take care of. In the aspect of preliminary PM_2.5_, the building industry released considerable amounts of particle matters during the construction process, and the road dust took significant responsibility. In terms of the PM_2.5_ emissions during energy consumption, electricity and heat supply accounts for a similar ratio in the transportation sector. It can be also observed that other industries have an important role in the emissions, which include the primary industry, the remainder of the secondary industry, and the rest of the tertiary industry. The detailed data can be consulted in Table A1 of the Annex.

**Figure 4:**
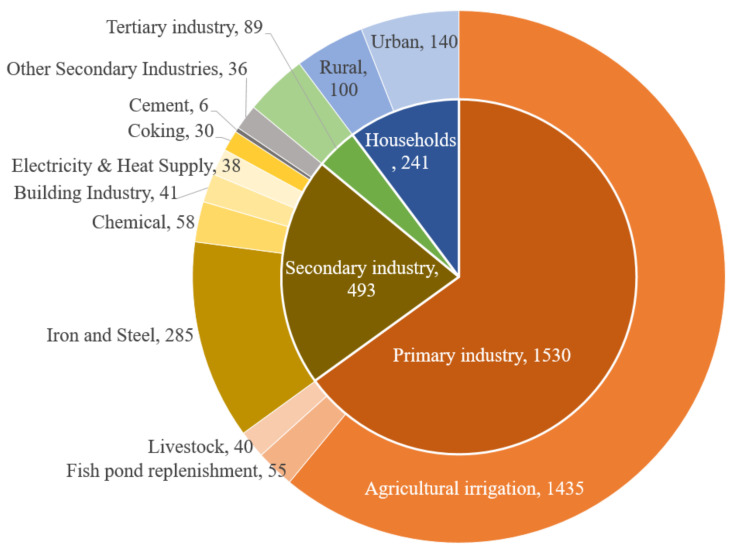
The sectoral water consumption of Tangshan in 2018 (million m^3^).

According to the Tangshan Agriculture and Rural Affairs Bureau and Tangshan Statistics Bureau, 2379 million m^3^ of freshwater were consumed in 2018, and the sectoral consumption is shown in Figure 4. A total of 64.3% of water went to the primary industry, by which the majority were utilized for agriculture irritation, and less than 4% of the water was used to replenish ponds and to feed livestock. The secondary industry accounts for about 21% of the total water consumption, and the iron and steel industry consume the majority. The chemical industry, building industry, electricity and heat supply, and coking industry consumed 30–58 million m^3^ of freshwater individually. The consumption by households was over 50% of the secondary industry level.

## 3. Scenario Design

The scenario analysis method combines the quantitative and qualitative predicting methods that are based on the information of relevant development strategies. It is a powerful tool to forecast future visions and the corresponding measures to reach the objectives, thus helping policymakers to understand better the direction of development.

The paper discussed two scenarios, which are termed as reference scenario (REF) and green low-carbon scenario (GLC), whose detailed measures are provided in Table 2. The REF scenario is based on the relevant policy documents issued by the central government, the Hebei Province, and Tangshan City. With the guidance of the *ACTION PLAN FOR CARBON DIOXIDE PEAKING BEFORE 2030* issued by the Chinese government in October 2021, all the measures are either source types or process types. Five categories are selected: economic structure adjustment, electricity and heat supply, critical high consumption and high-emission industries (iron and steel, cement, and chemical industries), transportation, and building. These correspond to 20 measures in total. The REF scenario is based on current policy and the preliminary carbon emission plans to control the development of high-pollution and high-consumption industries. The power supply system is required to decarbonize rapidly, energy efficiency is expected to increase in all the related sectors, and the energy consumption mix is also anticipated to adjust; the transportation system will undergo a systematic transformation toward a low carbon and green direction. The GLC scenario takes all the measures, but with a much more ambitious target, with the expectation to improve air quality and achieve the carbon emission peak in advance.

In terms of predicting the socio-economic development, the city’s five-year plan and 2035 long-term goal served as a significant reference. The main assumptions of GDP, population, and urbanization trends are given in Table 3, which combines the corresponding evolution history and the communications with the Tangshan Development and Reform Commission.

## 4. Results

### 4.1. Energy Consumption

**Figure 5:**
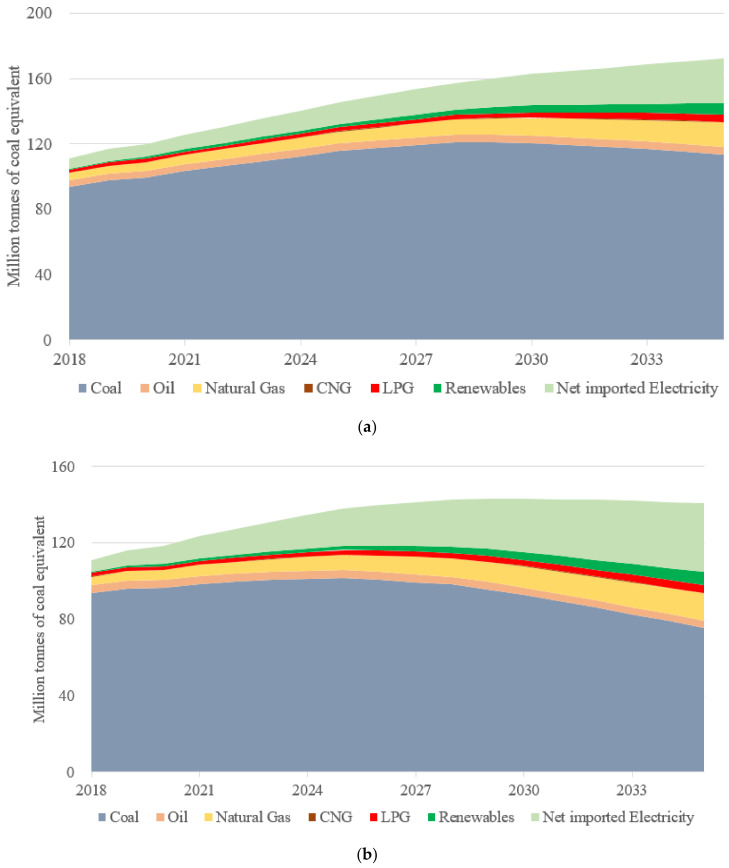
The predictions of city’s energy consumption. (**a**) REF scenario. (**b**) GLC scenario.

The energy consumption is expected to rise by the end of 2030 under both scenarios, as shown in Figure 5. However, it will continue to increase in the REF scenario, and the GLC scenario will achieve the energy consumption peak by 2030 with around 143.2 Mtce; from then on, the economic development will decouple with the energy consumption. Coal consumption will peak by 2029 and 2025 for REF and GLC, respectively, and increase by 29.4% and 8.5%, respectively, compared with the base year. Renewables will take a more important role in the GLC scenario since they will receive more incentives, as shown in Table 3. Natural gas will contribute more to the energy mix, expanding from 4% in 2018 to 8.7% and 10.2% in 2035 for GLC and REF, respectively. There are mainly two reasons that lead to the rapid rise of net imported electricity. The first one is the transition toward a green and low-carbon energy consumption structure, in which electrification is one of the most important means, and the total electricity consumption will soar. The second reason lies in the fact that electricity generation from coal power plants will be limited to a certain level; the added electricity generation from fossil fuels and renewables cannot meet the electricity balance, and the gap will become larger. Thus, the net imported electricity will witness obvious growth in the coming years for both scenarios, which will increase by 351% and 491% for REF and GLC scenarios, respectively, until 2035.

### 4.2. CO_2_ Emission

**Figure 6:**
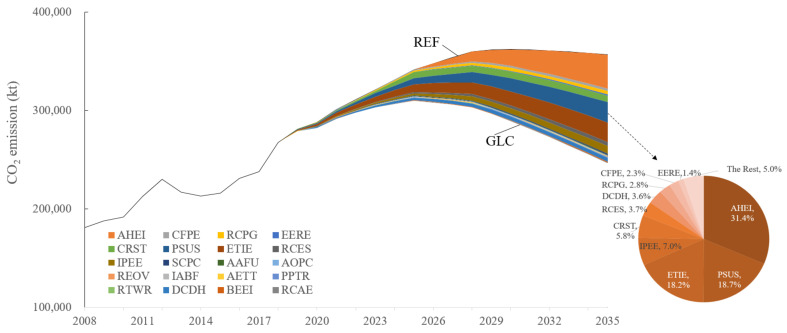
Predictions of CO_2_ emission and contribution of various policies from REF to GLC.

The simulation of the CO_2_ emissions of the REF and GLC scenarios are illustrated in Figure 6. The results show that the current plan is able to mitigate CO_2_ emissions and achieve the CEP in 2030 for the REF scenario, which will release 362.87 Mt. However, achieving the CEP at around 2030 might not be safe when unexpected things happen, and it is more prudential to achieve it earlier. The GLC scenario enforces stricter policies and is expected to reach the CEP around 2025, which will release 310.09 Mt. Taking the year 2035 as an example, AHEI illustrates the best abatement capability (31.4%), PSUS and ETIE contributes similar reductions (18.7% and 18.2%), and the top seven measures account for more than 90% of emission mitigation.

**Figure 7:**
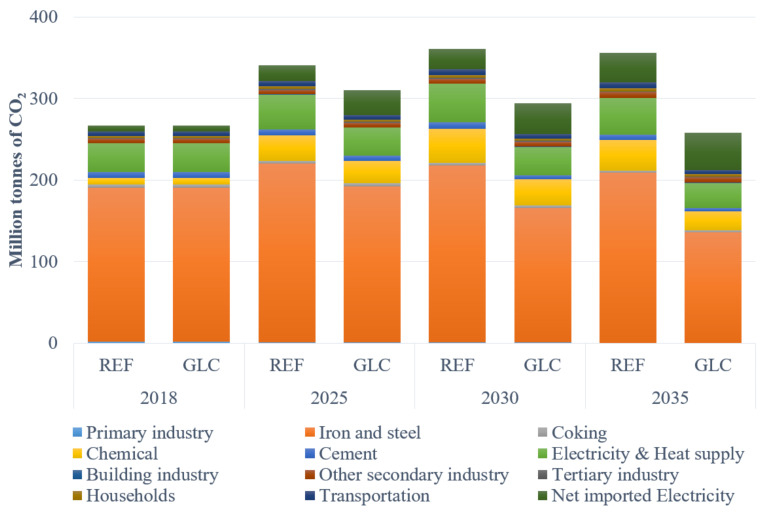
Prediction of CO_2_ emissions from critical sectors.

Looking from the sectoral CO_2_ emissions, as provided in Figure 7, the iron and steel industry still takes the first responsibility for the emissions, but it will decrease remarkably under the GLC scenario. Due to the expected large-scale refineries that operate before 2025 in the Caofeidian District (Subordinate district of Tangshan), the CO_2_ emissions of the chemical industry shall increase considerably. The CO_2_ emissions from electricity and heat supply will decrease gradually under the GLC scenario for the control of coal consumption and the improvement of energy efficiency, but the emission in the REF scenario will increase by 20.2% and 34.2% in 2025 and 2030, respectively, compared with the base year. The indirect CO_2_ emissions from the net imported electricity augment substantially as with the trend of the net imported electricity.

### 4.3. Air Pollutants Emissions

**Figure 8:**
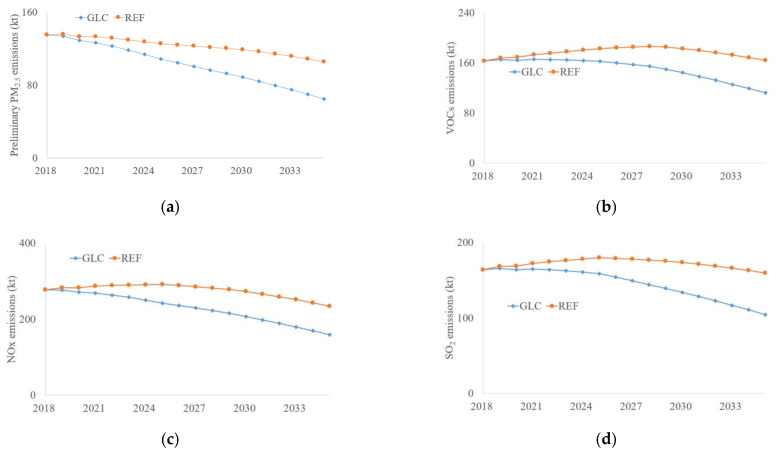
The main air pollutant emissions. (**a**) Preliminary PM_2.5_; (**b**) VOCs; (**c**) NOx; (**d**) SO_2_.

The air pollutant emissions vary differently for various types, See Figure 8. The preliminary PM_2.5_ emissions decrease continuously for both the REF and GLC scenarios; 21.9% and 51.8% of emissions will be reduced in 2035 for REF and GLC, respectively. The VOC emissions will increase before 2028 and then decrease for the REF scenario, but will still be higher than the base year level in 2035. The VOC emissions decrease slowly in the GLC scenario before 2024 and then enter into a rapid descent stage, as 31.2% of emissions will be abated by 2035. The NOx emissions increase slightly before 2025 for the REF scenario and finally achieve 15.7% of emission reductions in 2035, while the GLC scenario shows a smooth decrease in the coming years. The SO_2_ emission is similar to the trend of VOC emissions; REF scenario will increase the emissions by 9.6% and 5.9% in 2025 and 2030 compared with the base year, but 3.2% and 18.4% of emissions will be reduced under the GLC scenario for the same time periods, respectively.

### 4.4. Saved Water

**Figure 9:**
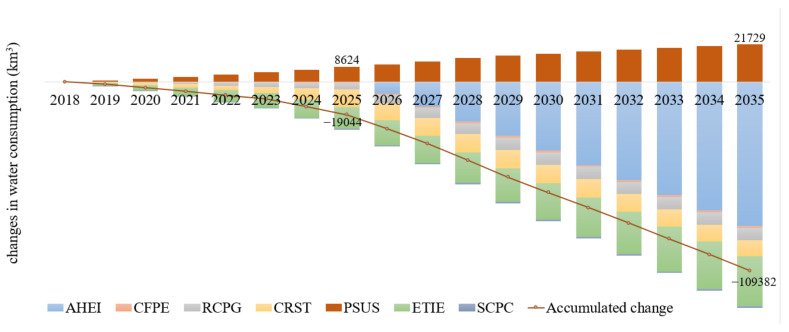
Water consumption variation from REF to GLC scenarios.

Implementing more ambitious policies to reach the goal of green and low-carbon development earlier allows for saving a significant amount of water, as presented in Figure 9. In 2035, the total effect of various policies will lead to 109.38 Mm^3^ of water saving, which corresponds to 4.6% of the current consumption. From REF to GLC, seven measures will influence the water consumption and six of them contribute to the saving, while PSUS requires more water during the transformation. Before 2025, the iron and steel industry and electricity and heat supply are the most critical sectors to affect water consumption. ETIE is the most efficient way to mitigate water consumption, followed by CRST and RCPG. After 2025, the adjustment of the economic structure in the GLC will accelerate, and low-carbon and high-efficiency industries will take more role in the economy. Since the tertiary industry and the new strategic industries require much less water per added value, AHEI will dominate the water-saving during the 16th five-year period. The transformation of blast furnace long process toward electric furnace short process has been regarded as a compulsory and urgent task in the iron and steel industry; it has a considerable potential to reduce air pollutant and CO_2_ emissions as shown above, but it consumes much more water per ton of crude steel. Thus, PSUS is the single policy that leads to the increase in water consumption, and it will offset about 20% of water savings.

### 4.5. Synergic Reduction Analysis

The synergic reduction analysis was carried out among CO_2_ emissions, air pollutant emissions, and water savings in the year 2030. *RSI* was utilized to reflect the synergic reduction ability of the selected policies toward a specific couple of targets, and SRC was drawn to rank the synergic capability of various policies. For all the measures listed in Table 3, EERE and IABF can abate CO_2_ emissions, but are unable to impact directly air pollutant emissions and water saving, and they are not included in the following discussions. The rest 18 measures influence at least two targets that are suitable to carry out the synergic study.

#### 4.5.1. VOCs and CO_2_ Emission

**Figure 10:**
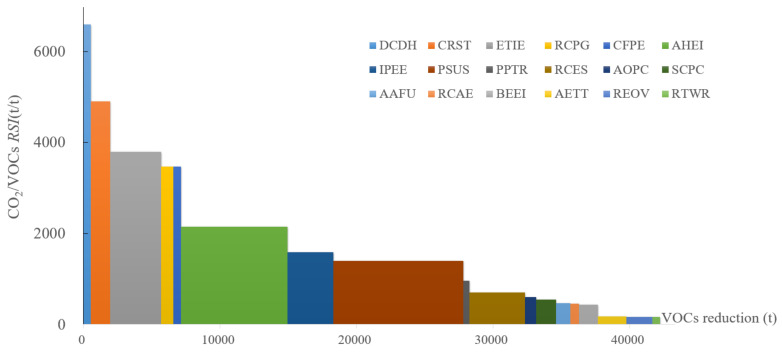
SRC of VOCs and CO_2_ emission in 2030.

The SRC between CO_2_ and VOC emission is provided in Figure 10. DCDH has the highest *RSI*, in which will abate 6959 units of CO_2_ when reducing one unit of VOCs. It also indicates that the district heating sectors can achieve the best synergic reduction. It is followed by CRST and ETIE, both in the iron and steel sector. RCPG and CFPE have an equivalent synergic effect since both of them affect the coal consumption in the coal power plant. RTWR has the lowest synergic reduction effect, with RSI being 163. From the VOC reduction ray, it can be read that PSUS allows for reducing the largest amount of VOCs (9529 t reduction potential); AHEI took the second place, followed by RCES and ETIE. In terms of the area of each block, PSUS, AHEI, and ETIE can reduce more CO_2_ in the total quantity factor. The accumulated VOCs reduction can be up to 42,236 t, which corresponds to 22.9% of the emission level in the REF scenario.

#### 4.5.2. PM_2.5_ and CO_2_ Emission

**Figure 11:**
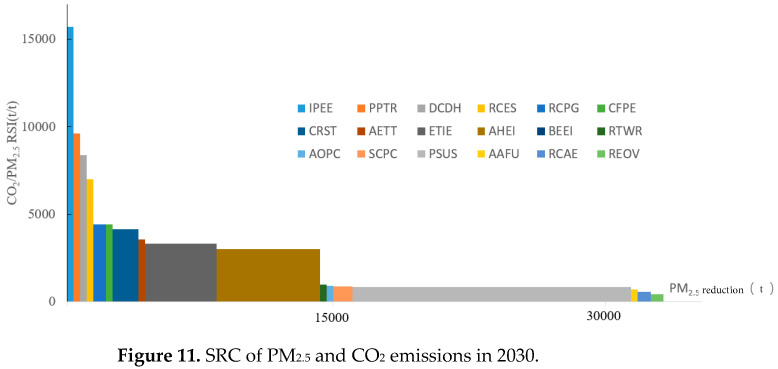
SRC of PM_2.5_ and CO_2_ emissions in 2030.

As shown in Figure 11, IPEE has the largest RSI between CO_2_ and PM_2.5_; 15,722 units of CO_2_ emission will be abated with the removal of one unit of PM_2.5_, followed by PPTR, DCDH, and RCES. Even though IPEE, PPTR, DCDH, and RCES have more than 5000 of RSI, their PM_2.5_ reduction capability is limited as the graph shows. The least synergic measure belongs to REOV, with the lowest RSI being 446. PSUS is the most powerful strategy to reduce PM_2.5_ reduction, with 15,698 t of reduction potential. AHEI and ETIE can reduce 5556 t and 4286 t of PM_2.5_, respectively. By implementing all the policies, 33,343 t of PM_2.5_ emission can be avoided, which means 28.2% of the emission in the REF scenario will be reduced.

#### 4.5.3. NO_x_ and CO_2_ Emission

**Figure 12:**
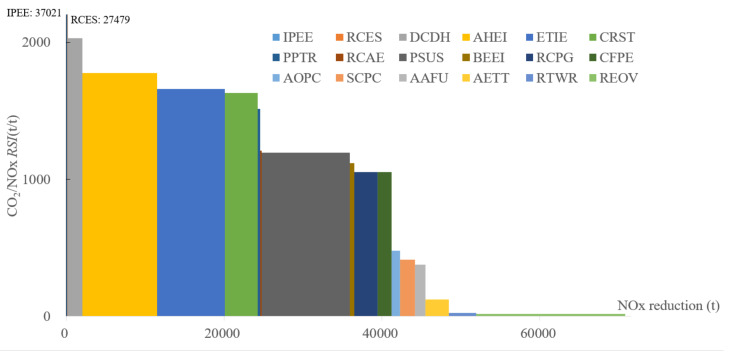
SRC of NO_x_ and CO_2_ emissions in 2030.

Most measures can achieve the positive synergic reduction in NOx and CO_2_ emissions as illustrated in Figure 12. IPEE and RCES have more than 10 times RSI compared with the rest of the measures. From left to right, the RSI between NO_x_ and CO_2_ of DCDH, AHEI, ETIE, CRST, and PSUS decreases gradually, but their impact on NO_x_ emission reduction cannot be neglected. It deserves to be noticed that, even though REOV has the greatest potential to mitigate NO_x_ emissions (18,846 t), it has the lowest RSI. It means that the policy in the transportation sector needs to highlight the NO_x_ emission reduction. The total NO_x_ emission reduction capability is 70,796 t, which equals to 26.0% of the emission levels in the REF scenario.

#### 4.5.4. SO_2_ and CO_2_

**Figure 13:**
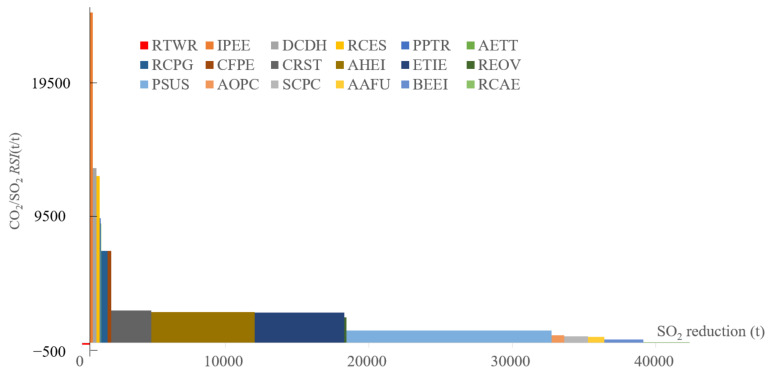
SRC of SO_2_ and CO_2_ emissions in 2030.

The SRC result between SO_2_ and CO_2_ is slightly different from the above SRC results since RTWR is located in the third quadrant, as illustrated in Figure 13. RTWR aims to prompt the railway and water transportation to replace the road freight transportation; it will increase SO_2_ emission since ships consume more fuel oil and the sulfur emission factor is larger than the diesel combusted in trucks. The increasing potential will be 548 t of SO_2_. Except for RTWR, 17 measures can reduce CO_2_ and SO_2_ emissions at the same time. IPEE has the largest RSI (over 26,000); DCDH, RCES, PPTR, and AETT also have very high RSI behind IPEE, while they have a rather limited capability to abate SO_2_ emissions. Even though PSUS has a relatively low RSI between SO_2_ and CO_2_, it is the most promising measure to reduce SO_2_ emissions, with 14,286 t potential to remove them. CRST, AHEI, and ETIE remove less SO_2_ than PSUS, but their RSI will double. The accumulated SO_2_ emission can be up to 44,534 t, which corresponds to 25.4% of the emission level in the REF scenario.

#### 4.5.5. Air Pollutant Equivalent and CO_2_ Emission

To analyze the comprehensive reduction in air pollutants, the air pollutant equivalent *AP_eq_* was employed to add up the emissions of PM_2.5_, NO_x_, VOCs, and SO_2_ by Equation (12), where $\Delta E_{i}^{s}$ stands for the specific *i* air pollutant emissions by policy *s*, $W_{i}$ indicates the pollution equivalent value recommended by sewage tax from the Chinese government as provided in Table 4, and *i* means the four air pollutants.

(12) $$\Delta AP_{eq}^{s}\;=\;\Delta E_{PM_{2.5}}^{s}/W_{PM_{2.5}}\;+\;\Delta E_{NOx}^{s}/W_{NOx}\;+\;\Delta E_{VOCs}^{s}/W_{VOCs}\;+\;\Delta E_{SO_{2}}^{s}/W_{SO_{2}}$$

**Figure 14:**
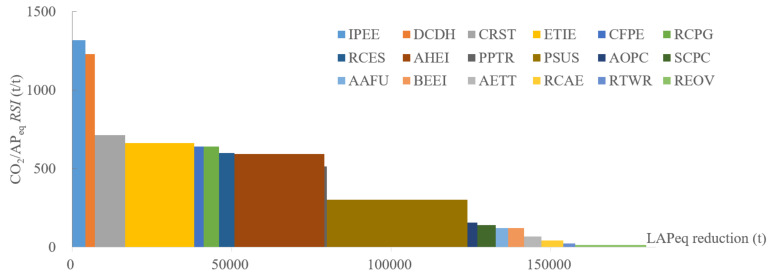
SRC of AP_eq_ and CO_2_ emissions in 2030.

As presented by Figure 14, the highest RSI between *AP_eq_* and CO_2_ belong to IPEE, in which the energy efficiency improvement in the chemical industry exhibits the best synergic reduction ability in terms of the total quantity. The second-best RSI is RCES, which also belongs to the chemical industry. The measures in the iron and steel industry also have a considerable synergic reduction ability, as it can be observed that CRST and ETIE follow closely with RCES, but they have much better capability to remove air pollutants. In addition, the largest potential to reduce equivalent air pollutants is policy PSUS, by transforming the traditional steel production process toward an electric process. AHEI contributes to the air pollutant reduction just behind PSUS, but with a double RSI to achieve a much better synergic mitigation ability.

#### 4.5.6. Water Saving and CO_2_

**Figure 15:**
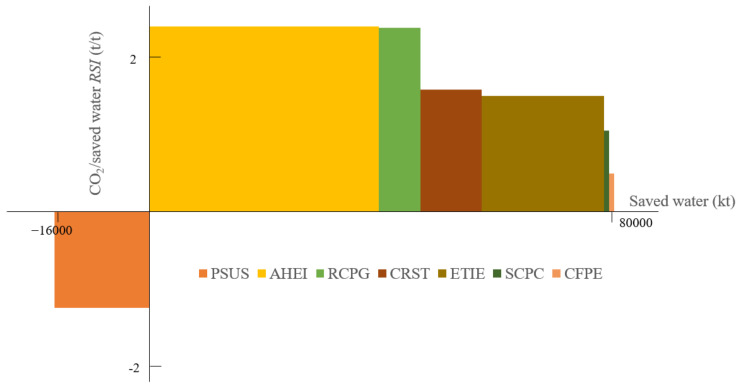
SRC of water saving and CO_2_ emissions in 2030.

The synergic effect of water saving and CO_2_ emission reductions is given in Figure 15. Seven measures influence water consumption and CO_2_ emission at the same time, and PSUS leads to the increase in consumption, as discussed in part 4.4. The remaining measures are located in the first quadrant, and the saved water quantity is close to the CO_2_ mitigation since all the RSIs are in the range between −1.5~2.5. Quite different from the above SRC between air pollutants and CO_2_, the policy AHEI has the highest RSI between CO_2_ reduction and water saving and is also the one that achieves the majority of water saving (39,768 t). The RSI of RCPG is slightly smaller than AHEI, but the water-saving capability is far lower. It is also interesting to note that the SRI of SCPC and CFPE is in the last position, and their water-saving potential is also in the same situation. In other words, maintaining a strategic determination to adjust the economic structure is the most efficient way to save water and reduce CO_2_ emissions synergistically. The efficiency improvement in coal power plants and controlling the cement production cannot contribute significantly to water saving. The GLC scenario enables to save 6422 m^3^ of water consumption, which represents 2.3% of water consumption in the REF scenario.

### 4.6. Multi-Objective Reduction Analysis

**Figure 16:**
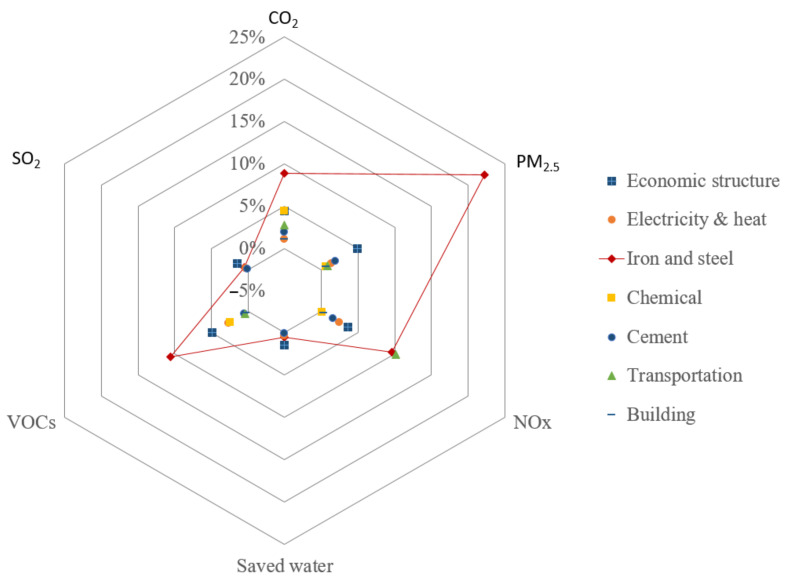
Sectoral reduction degree of multi-objective.

Exploring the sectoral contribution toward multi-objective, as discussed above, a radar graph can help to visualize the reduction degree potential of each sector, as illustrated in Figure 16. Iron and steel, as the major energy consumption and emission sector, have the largest reduction degree potential in CO_2_, PM_2.5_, and VOCs, reaching to 8.9%, 22.3%, and 10.6%, respectively. Adjusting the economic structure can have the best reduction degree in SO_2_ and water saving, and the performance in the reduction in VOCs, PM_2.5_, and NO_x_ is also good. The transportation sector leads a NO_x_ reduction and is slightly higher than the iron and steel sector, which is significantly higher than that of other sectors. The chemical industry has a good performance in the reduction in CO_2_ and VOCs. The building sector ranks almost last place in all the objects since the fossil energy consumption is quite smaller than other sectors.

Table 5 provides the ESI of CO_2_–air pollutant emissions and CO_2_ emission–water consumption of the proposed measures in 2030 under the GLC scenario. By adjusting the economic structure, AHEI has a promising synergic reduction capability (ESI > 1), except that the ESI of PM_2.5_ is slightly lower than 1. CFPE and RCPG in the electricity and heat supply sector also show a good synergic reduction performance with most of ESI > 1 in addition to the NO_x_. CRST and ETIE in the iron and steel sector have a significant synergic reduction potential with all the ESI > 1. The synergic reduction capability of PSUS in the air pollutants is good (0.25 < ESI < 0.92), but it also has a negative correlation (ESI < 0). In the chemical industry, RCES and IPEE have quite good synergic reduction capability in PM_2.5_, NO_x_, and SO_2_ (ESI > 2). The three measures in cement production have a good synergic reduction capability (0.21 < ESI < 0.37) for air pollutants and the ESI of SCPC in the aspect of water saving is quite high (ESI > 1). In the transportation sector, the synergic reduction potential of REOV, AETT, and RTWR in NO_x_ and VOCs is limited (0 < ESI < 0.1). AETT and PPTR have quite a good synergic reduction potential in PM_2.5_ and SO_2_. However, the ESI of RTWR in SO_2_ is negative. In the building factors, DCDH has a remarkable synergic reduction potential in air pollutants (ESI > 1.5), and the capabilities of BEEI and RCAE are also good (0 < ESI < 1). In terms of the synergic reduction degree of an individual object, IPEE has the highest ESI in both PM_2.5_ and NO_x_, and the largest ESI of VOCs, SO_2_, and APeq is occupied by DCDH. CFPE has the highest ESI in water.

## 5. Conclusions

The paper aimed to explore the air pollutants–CO_2_-water nexus under the CEP target, with the expectation to achieve synergic benefits by carrying out more ambitious measures to transform social and economic development. Tangshan is a representative city in the Jing-Jin-Ji region with iron and steel as the key industry for social development. The LEAP model was employed to predict energy consumption and CO_2_ and air pollutant emissions. The REF and GLC scenarios were constructed to forecast future conditions. The two scenarios share the same policies, which are all under the guidance of the carbon emission peak plan and also the relevant plan of local government, ranging from the economic structure adjustment, transformation of electricity and heat supply, secondary industry, transportation, and building industry. Twenty measures were involved, where GLC takes a more aggressive target in every measure based on REF. Differentiating from the usual study, the REF scenario is designed to achieve the CEP before 2030 to make the business-as-usual scenario more persuasive under the current development targets. The SRC was proposed to illustrate the co-benefits between CO_2_ emission mitigation and air pollutants, and saved water. Compared with a commonly utilized tool to include them in the coordinate system, it can exhibit a straightforward synergic reduction capability, and the reduction potential in the quantity aspect is more direct. Synergic reduction elasticity was also employed to compare various objects in the reduction degree aspect. Under the GLC scenario, the carbon emission peak can be achieved in 2025. By 2030, air pollutant emissions will decrease around 25% for the PM_2.5_, NO_x_, SO_2_, and VOCs; the water consumption is expected to reduce by 2.3%; and CO_2_ abatement will be 19.9%, which illustrate a considerable synergic reduction potential. The decarbonization of district heating exhibits the best synergic reduction capability between SO_2_ and CO_2_. Energy efficiency improvement in the chemical industry performs the best synergic reduction capability between PM_2.5_ and CO_2_, NO_x_ and CO_2_, and also SO_2_ and CO_2_. Accelerating the adjustment of economic structure saves the most water, reduces the largest amount of CO_2_ emission, and also has the best synergic reduction capability between water consumption and CO_2_. Transforming the traditional long process steelmaking toward short process electric has the largest quantitative capability to reduce PM_2.5_, SO_2_, and VOC emissions, even though it has a considerable water consumption increase. The largest reduction in NO_x_ can be expected by retiring old vehicles.

The following policy suggestions are provided based on the above analysis:

- Adopting ambitious measures enables the city to obtain more synergic benefits. The CEP-plan-related measures are source-type governing choices, which can usually lead to positive synergic benefits environmentally. To make the targets stronger, less air pollutants and CO_2_ will be emitted, and a lower water consumption is required. However, it also ought to carefully set the targets to make sure it is bearable and does not violate the safety of supply chains;
- Sticking tirelessly to promote economic restructuring. Economic adjustment is the most efficient way to remove CO_2_ and air pollutant emissions and water consumption through the transition toward strategic emerging industries and low-carbon and high-efficiency industries;
- Energy efficiency improvements ought to be online all the time. As illustrated in the results, energy efficiency improvement in industries plays a significant role in reducing air pollutants, CO_2_, and water consumption. The synergic reduction ability is also of priority. Improving the energy efficiency can bring synergic benefits continuously;
- End-of-pipe measures might still be necessary for controlling air pollutants. Even though all the above-mentioned policies can reduce air pollutants significantly (20~30%), they still exist in large quantities. To deeply improve the air quality, strict emission standards ought to be carried out to further decrease air pollutants;
- Being careful about iron and steel transformation. Controlling the product yield and improving energy efficiency contribute to positive synergic benefits. However, the electric steelmaking process consumes much more water than the traditional technique. The transformation pathway can be optimized by considering the relevant air pollutants, CO_2_ emissions, and water consumption requirements;
- The transportation sector requires specific attention. The transformation of freight transport from the road toward waterways might increase SO_2_ emissions, due to the higher emission factor of ships. It might be necessary to increase the emission standard of the fuel oil quality and also the emission standards of fuel engines to avoid a negative synergic effect.

The research enlarges the topic of the synergic control of air pollutants and GHG emissions by proposing a method to estimate water-saving potential when achieving the CEP. It is also worth noticing that the work can be further improved in two aspects: one is to optimize the carbon emission plan by carrying out a cost–benefit analysis together with the net value optimization, and the other one is to apply the synergic benefit study to wider domains by discussing solid waste, soil pollution, among others.

## Figures and Tables

**Table 1:** The structure of LEAP-TS.

Sectors/Sub-Sectors	Activity Level		Energy Structure
Primary industry	Added value		Anthracite, bituminous, hard coal briquettes, diesel, electricity
Secondary industry	Iron and steel	Added value	Bituminous, coke, other washed coal, coke oven gas, natural gas, diesel, electricity
	Petroleum-processing coking industry	Added value	Bituminous, electricity, diesel, coke oven gas
	Chemical industry	Added value	Bituminous, coke, other washed coal, coke oven gas, natural gas, diesel, electricity, heat
	Building materials	Added value	Bituminous, natural gas, electricity, biomass, municipal sewage sludge
	Other parts of the secondary industry	Added value	Bituminous, coke, coke oven gas, LPG, heat
	Building industry	Added value	Bituminous, diesel, fuel oil, electricity
	Electricity and heat supply	Added value	Bituminous, natural gas, biomass, wind, solar, domestic waste
Tertiary industry	Service industry	Added value	Bituminous, diesel, natural gas, LPG, electricity, heat
	Transportation: passengers	Number of vehicles, mileage	Gasoline, diesel, CNG, electricity
	Transportation: freight	Freight turnover	Gasoline, diesel, CNG, electricity, biofuels
Households	Urban	Population	Bituminous, natural gas, electricity, heat, LPG
	Rural	Population	Bituminous, natural gas, diesel, electricity, LPG

**Table 2:** Scenario settings based on the carbon emission peak plan.

Sector/Industries	Reduction Policies/Measures	Reference Scenario (REF)	Green Low Carbon Scenario (GLC)	Abbreviation
Economic Structure Adjustment	1. Accelerating the development of strategic emerging industries and low-carbon and high-efficiency industries	By 2025, the proportion of the secondary industry will drop to 50%. By 2035, the proportion of the secondary industry will drop to 45%, and the proportion of low-carbon and high-efficiency industries in the secondary industry will increase by 10 percentage points	By 2025, the proportion of the secondary industry will drop to 50%. By 2035, the proportion of the secondary industry will drop to 40%, and the proportion of low-carbon and high-efficiency industries in the secondary industry will increase by 30 percentage points	AHEI
Electricity and heat supply	2. Coal-fired power plant efficiency improvement	By 2035, coal consumption per kWh electricity generated will reduce 10% compared with the base year	By 2035, coal consumption per kWh electricity generated will reduce 20% compared with the base year	CFPE
	3. Regulation of coal power generation	By 2035, the electricity generated by coal power plant will increase no more than 50% compared with the base year	By 2035, the electricity generated by coal power plant will increase no more than 30% compared with the base year	RCPG
	4. Increasing the electricity generation by photovoltaic energy, wind power and biomass	By 2035, the electricity generated by photovoltaic energy and wind will increase by 5 and 12, respectively	By 2035, the electricity generated by photovoltaic energy and wind will increase by 10 and 14 times, respectively	EERE
	5. Decarbonization of district heating	By 2035, 15% of the district heating will be provided by renewable energy or industrial waste heat	By 2035, 30% of district heating will be provided by renewable energy or industrial waste heat	DCDH
Iron and steel	6. Strictly control of crude steel output	The production of crude steel will peak until the end of 2025, about 20% higher than the base year	The production of crude steel will peak around 2023, about 15% higher than the base year	CRST
	7. Adjust the process structure and increase the utilization of scrap steel	By 2035, the proportion of crude steel production using scrap steel short process will increase to 10%	By 2035, the proportion of crude steel production using scrap steel short process will increase to 20%	PSUS
	8. Energy-saving technical transformation to improve energy efficiency	By 2035, the energy consumption per CNY 10,000 of added value will decrease by 7%	By 2035, reduce energy consumption per 10,000 yuan of added value by 15%	ETIE
Chemical Industry	9. Reducing coal consumption and adjust energy structure	By 2035, the proportion of coal consumption will decrease by 10%, the proportion of natural gas consumption will increase by 5%, and the proportion of electricity consumption will increase by 5%	By 2035, the proportion of coal consumption will decrease by 20%, the proportion of natural gas consumption will increase by 15%, and the proportion of electricity consumption will increase by 10%	RCES
	10. Improving energy efficiency	By 2035, the energy consumption per CNY 10,000 of added value will reduce 15%	By 2035, the energy consumption per CNY 10,000 of added value will reduce 30%	IPEE
Cement production	11. Strictly control the production of cement clinker	The production of cement clinker will peak at the end of the 14th Five-Year Plan	The production of cement clinker will peak in the early period of the “14th Five-Year Plan”	SCPC
	12. Accelerating alternative fuel utilization	By 2035, coal consumption will decrease 8%	By 2035, coal consumption will decrease 15%	AAFU
	13. Accelerating the elimination of outdated production capacity and promote energy efficiency improvement technologies	By 2030, eliminate all 2000 t/day production lines, and production lines with energy efficiency levels that are 30% or more higher than the national leading value; by 2035, energy consumption per CNY 10,000 of added value will reduce by 7%	By 2030, eliminate all 2000 t/day production lines, and production lines with energy efficiency levels that are 30% or more higher than the national leading value; by 2035, energy consumption per CNY 10,000 of added value will reduce by 15%	AOPC
Transportation	14. Retiring old vehicles	Eliminating all yellow-label cars and old cars by 2025, and phasing out all the cars with emission standards below national 4 by 2035	By 2025, eliminating all the cars with emission standards below national 3, and phasing out those below national 5 by 2035.	REOV
	15. Increasing the application of biofuels	By 2035, the proportion of biofuel oil consumption in the freight transportation will reach 4%	By 2035, the proportion of biofuel oil consumption in the freight transportation will reach 10%	IABF
	16. Accelerating the electrification of transportation tools	By 2035, the electrification rate of buses, private cars and freight vehicles will reach 80%, 10% and 10%, respectively	By 2035, the electrification rate of buses, private cars and freight vehicles will reach 100%, 20% and 20%, respectively	AETT
	17. Promoting public transport	By 2035, the city’s public transport trip share rate will increase 10 percentage points	By 2035, the city’s public transit trip share rate will increase by 20 percentage points	PPTR
	18. Promote the transition of road transportation toward water and railway transportation	By 2035, the railway and water freight turnover will increase by 5% and 10%, respectively	By 2035, the railway and water freight turnover will increase by 10% and 15%, respectively	RTWR
Building	19. Building energy efficiency improvement	By 2035, the energy consumption per unit building area will decrease 10%	By 2035, the energy consumption per unit building area will decrease 20%	BEEI
	20. Reducing the proportion of coal consumption and accelerate the electrification process	By 2035, the electricity in the energy mix will increase 10 percentage points, and the proportion of coal consumption will decrease 10 percentage points	By 2035, the electricity in the energy mix will increase 20 percentage points and the proportion of coal consumption will decrease 20 percentage points	RCAE

**Table 3:** Prediction of socio-economic parameters.

	2021–2025	2026–2030	2031–2035
Annual GDP increase	5.5%	5%	4.5%
Annual population growth	0.4%	0.2%	0.1%
Annual urbanization growth	1.03%	1.0%	1.0%

**Table 4:** Air pollutant pollution equivalent values [43].

Air Pollutants	Pollution Equivalent Value
PM_2.5_	4
SO_2_	0.95
NO_x_	0.95
VOCs	0.95

**Table 5:** ESI of CO_2_–air pollutants and CO_2_–water by the proposed measures in 2030.

Sector	Measures	PM_2.5_	NO_x_	VOCs	SO_2_	AP_eq_	Water
Adjust economic structure	AHEI	0.987	1.356	1.090	1.123	1.183	3.349
Electricity and heat supply	CFPE	1.455	0.790	1.767	3.378	1.275	15.796
	RCPG	1.452	0.791	1.763	3.367	1.274	3.242
	EERE	-	-	-	-	-	-
	DCDH	2.763	1.534	3.344	6.306	2.439	-
Iron and steel	CRST	1.364	1.249	2.518	1.181	1.432	5.183
	PSUS	0.253	0.913	0.710	0.432	0.594	−6.815
	ETIE	1.100	1.280	1.991	1.110	1.342	5.670
Chemical industry	RCES	2.328	21.413	0.354	6.218	1.206	-
	IPEE	5.210	28.651	0.805	12.727	2.651	-
Cement production	SCPC	0.285	0.315	0.277	0.235	0.278	7.656
	AAFU	0.230	0.287	0.236	0.206	0.244	-
	AOPC	0.300	0.364	0.307	0.265	0.313	-
Transportation	REOV	0.146	0.012	0.083	0.932	0.027	-
	IABF	-	-	-	-	-	-
	AETT	1.173	0.092	0.088	4.355	0.134	-
	PPTR	3.176	1.141	0.488	4.540	1.019	-
	RTWR	0.316	0.020	0.082	−0.080	0.048	-
Building	BEEI	0.812	0.843	0.218	0.104	0.237	-
	RCAE	0.180	0.911	0.229	0.024	0.081	-

## Data Availability

Not applicable.

## Appendix A

**Table A1:** The sectoral contribution of CO_2_ and air pollutant emissions of Tangshan in 2018.

	CO_2_	SO_2_	NO_x_	VOCs	Preliminary PM_2.5_
Electricity and heat supply	12.0%	1.5%	6.5%	4.0%	4.0%
Iron and steel	64.1%	56.9%	43.7%	34.4%	50.0%
Cement	2.3%	8.5%	5.8%	7.7%	6.2%
Chemical	2.9%	0.3%	0.2%	3.7%	0.5%
Building industry	0.1%	-	-	-	20.3%
Households and service industry	1.2%	17.4%	1.5%	11.3%	2.0%
Other industries	15.5%	10.2%	8.8%	29.1%	13.8%
Transportation	1.9%	5.3%	33.6%	9.7%	3.4%
